# Pecking Behavior in Conventional Layer Hybrids and Dual-Purpose Hens Throughout the Laying Period

**DOI:** 10.3389/fvets.2021.660400

**Published:** 2021-04-23

**Authors:** Lorena Rieke, Birgit Spindler, Isabel Zylka, Nicole Kemper, Mona Franziska Giersberg

**Affiliations:** ^1^Institute for Animal Hygiene, Animal Welfare and Farm Animal Behavior, University of Veterinary Medicine Hannover, Foundation, Hanover, Germany; ^2^Animals in Science and Society, Department Population Health Sciences, Faculty of Veterinary Medicine, Utrecht University, Utrecht, Netherlands

**Keywords:** laying hen, aviary, behavior, welfare, feather pecking

## Abstract

To avoid the killing of surplus male layer chickens, dual-purpose hybrids are suggested as an alternative approach. These strains may offer additional advantages compared to conventional laying hens, for instance, a lower tendency to develop injurious pecking behavior. The aim of this study was to assess the behavior, with focus on pecking behavior, of conventional layers (Lohmann Brown plus, LB+) and dual-purpose hens (Lohmann Dual, LD). About 1,845 hens per strain with intact beaks were housed in four stable compartments in aviary systems. Video-based scan sampling of general behaviors and continuous observations of pecking behavior were carried out between 25 and 69 weeks of life. With the exception of “dustbathing” and “scratching,” hybrid × time during the laying period affected all of the observed general behaviors [*F*_(2, 89)_ = 3.92–10.81, *P* < 0.001–0.05]. With increasing age, the LB+ hens performed more general pecking, more locomotion and less comfort and sitting behavior. General pecking and comfort behavior did not change over time in the LD hens, whereas inactive behaviors increased with age. During continuous observations, a significant hybrid x period interaction was found for all forms of pecking behavior [*F*_(2, 89)_ = 4.55–14.80, *P* < 0.001–0.05]. The LB+ hens showed particularly more severe feather pecking (SFP), which increased with age. In contrast, SFP remained exceptionally low in the LD hens throughout production. Therefore, dual-purpose hybrids should be considered as an alternative to both avoid the killing of surplus male chickens and the development of SFP in laying hen production.

## Introduction

Killing male day-old chickens from layer strains directly after hatch is a common practice all over the world. These chickens are not suitable for economic meat production due to the genetically determined negative correlation between fattening and reproductive performance (1). This led to the selection of specialized hybrid strains for either meat production (broiler hybrids) or egg production (layer hybrids). In broiler hybrids, both sexes are used for meat production. However, as male layer hybrids do not lay eggs, they are usually killed at day-old. This practice raises strong socio-ethical—and in some European countries—also legal concerns (2). Thus, in recent years, different approaches to avoid this practice have been developed (3). A dual-purpose concept that consists of housing hens for egg production and keeping roosters for meat production, is one solution to deal with the problem of surplus male chickens of very specialized hybrid strains. According to a public survey, dual-purpose chickens seem to be one of the preferred alternatives (4). Other approaches include different methods of in-ovo sex determination, with discharging eggs with male embryos at a preferably early stage of incubation and only hatching female chickens (2, 3). In other cases, the male offspring of conventional, high-yielding layers are reared without economic profit. Their meat is mainly processed for convenience food and the costs of rearing are usually subsided by a higher price for the eggs from such concepts (2). However, these concepts do not address additional challenges in the context of animal welfare in modern laying hen husbandry. For instance, the frequent occurrence of abnormal behaviors, such as injurious pecking (5, 6). In this respect, the use of dual-purpose hens may also provide advantages compared to conventional layers, as they seem to show a lower tendency to develop injurious pecking behavior (6).

By the term “injurious pecking,” several behaviors are summarized, which are all directed at conspecifics and lead to physical damage. Feather pecking (FP) is a form of injurious pecking with multifactorial origins, posing a serious welfare threat to current laying hen husbandry (5, 7, 8). This undesirable behavior impairs the health and welfare of the animals as it causes pain in the receiver and is a sign of stress in the offending bird (9). FP refers to non-aggressive pecking, and is directed mainly at the bird's back, tail, and vent area (5, 10). Different forms of FP can be distinguished depending on the forces of the pecks, and whether feathers are completely removed or not (11). A distinction between severe feather pecking (SFP) and gentle feather pecking (GFP) is suggested, as SFP causes most of the feather damage to the recipient bird (10). SFP is characterized by forceful pecks that result in feathers being pulled out, accompanied by a reaction of the recipient bird. In contrast, GFP normally does not result in feather loss or a reaction from the receiver (5). Nevertheless, GFP may develop into SFP, which itself can turn into tissue pecking and cannibalism as soon as denuded areas occur on the hens' bodies (11). In contrast, cannibalistic behavior that is directed at the vent or the toe of a conspecific often occurs irrespectively of FP in hens with intact plumage cover (11–13). Moreover, feather pecking must be distinguished from aggressive pecking (AP), as the latter occurs due to a different underlying motivation (5). AP is regarded as a normal dominance behavior to establish social hierarchies. It is mainly directed at the birds' head and neck, and usually does not result in severe feather damage (5, 11). More recent research suggests that FP behavior is an over-expression of social exploration (7). However, FP has also been considered as redirected foraging behavior (14). In this respect, the inhibition of environmental pecking (EP) at the ground or other surfaces in the barn may cause a redirection of pecking at the bodies of conspecifics (14, 15). Besides foraging, which includes EP and scratching, there are other, more general behaviors that influence or are influenced by FP behavior. Similarly to foraging, dustbathing behavior includes phases of EP. FP has also been associated with locomotion behavior. Hens selected for high levels of FP traveled longer distances (16) and showed higher levels of general locomotor activity (17) than birds selected for low FP activity. Comfort behavior is seen as a behavioral priority in laying hens (18) and its presence or absence can provide more general indications of the welfare status of a flock.

The likelihood of developing injurious pecking is influenced by many parameters such as resource-related factors. Resource-related factors may include the presence of dustbathing and foraging material, the arrangement of perches, the stocking density and the total amount of space provided (19–23). Thus, the prevention of abnormal behaviors seems mainly related to an optimization of husbandry and management conditions (8, 24). However, a correlation between FP and the genetic background of the hens was also previously described (7, 25, 26). Observational on-farm studies showed that the prevalence of feather damage varied among different commercial high-yielding layer strains (27, 28). Furthermore, a divergent phenotypic selection on FP behavior led to the high- and low FP chicken lines, which are used in fundamental research (26, 29). It was also possible to identify quantitative trait loci for FP behavior by using methods of molecular genetics (30). To date, little is known about the prevalence and the development of injurious pecking behavior in dual-purpose hens. In a previous longitudinal study, the plumage and integument condition of dual-purpose hens and conventional layer hybrids was comparatively assessed by a visual scoring method, indicating that severe feather loss and skin injuries were only present in the conventional layers but not in the dual-purpose hens (6). However, this research did not include behavioral results to support differences in actual FP activity between the two hybrids. Although feather loss and injuries are valid indicators for pecking behavior in laying hens (10), differences in plumage and integument condition might also be due to strain differences in feather quality or resource use causing more or less abrasion. So far, evidence that FP activity is higher in conventional layers compared to dual-purpose hens is only found in the functional area of the nest boxes (31). Thus, it is not known whether and to which extent the behavior, particularly injurious pecking behavior, differs between the two hybrid strains in other parts of the housing system.

The aim of the present study was to compare the pecking behavior and general behaviors potentially related to pecking behavior of conventional layer hybrids (Lohmann Brown plus, LB+) and dual-purpose hens (Lohmann Dual, LD) throughout the laying period (25–69 weeks of age). Based on previous research on feather loss and injuries, we hypothesized that the LD hens would show less injurious pecking behavior than the LB+ hens. Furthermore, we expected that within both hybrid strains, general behaviors and pecking behavior would be affected by age.

## Materials and Methods

### Animals and Husbandry

The present study involved a total of 3,690 Lohman Brown plus (LB+, conventional layer hybrid) and Lohman Dual (LD, dual-purpose hybrid) hens, all of them with untrimmed beaks. From day-old to 19 weeks of age, all LB+ and LD chickens were reared on a commercial farm in Northern Germany. The chickens were kept in the same house in one separate pen per hybrid (10 pullets/ m^2^). In both pens, the birds had unrestricted access to nipple drinkers (one nipple every 13 pullets), perches at different heights (35–95 cm above the floor, 30 m perching space/pen), wood shavings on the floor, and two straw bales. A commercial diet was provided *ad libitum* in pan feeders on elevated tiers (one feeder every 35 pullets). Wood shavings and straw bales were available in both pens on arrival of the chicks from the hatchery. A good litter quality (dry and flaky, easy to move with foot) was maintained in both pens during the entire rearing phase. All housing and management conditions were kept the same for the two hybrids (Supplementary Material) and the same caretaker looked after all pullets. Behavioral observations were not carried out during rearing. However, feather loss and injuries, which indicate feather pecking and cannibalism, were not observed in the LB+ and the LD pullets (Supplementary Material). At 19 weeks of age, the birds were transported to the research farm of the University of Veterinary Medicine, Hannover, Germany, where they were kept until the birds were 71 weeks of age. Again, both hybrids were subjected to the same standard housing and management conditions (32). The hens were housed in two stable compartments per hybrid (about 920 hens per compartment, 9 hens/m^2^) (31). Each compartment was equipped with six sections of an aviary system (Natura Nova 270, Big Dutchman, Vechta, Germany; total height: 200 cm) (33). The aviary was equipped with eight perches at four different heights (33–103 cm above a grid tier of 65 cm height) offering about 17 cm perching space per hen (33). In addition, the hens had access to linear feeding throughs (12.5 cm per hen), nipple drinkers (one nipple every 6.4 hens) and colony nest boxes (0.008 m^2^ per hen). On the floor, beneath and on both sides of the aviary, the hens had access to a scratching area with sawdust litter. Alfalfa bales suspended in hay nets served as standard enrichment material (about one bale every 200 hens). The light regime started with 10L:14D (week 19) and was gradually extended until 14L:10D (week 25). At 45 weeks, the light regime was increased to 16L:8D and maintained until the end of the laying period. At first signs of feather pecking or cannibalism additional measures, for instance pecking blocks, were placed in all stable compartments following a gradual emergency scheme (34). Detailed information on the emergency scheme, the measures being taken and general production data were reported by Giersberg et al. (6, 34).

### Behavioral Observations and Data Collection

For video-based data recordings, four cameras (EverFocus EQ610e, EverFocus Electronics Corp., Taipei, Taiwan) connected to a hard-drive recorder (EverFocus ECOR 264-9X1, EverFocus ElectronicsCorp., Taipei, Taiwan) were installed (one camera per stable compartment). Data were recorded for 1 day per week at three times (25th−30th, 43rd−48th, and 64th−69th week of life) during the laying period. These observation times were chosen based on a previous study (6), in which the onset of plumage loss around week 25, first injuries and severe plumage loss around week 43, and an exacerbation of the damage until the end of the laying period in the LB+ hens indicated the occurrence of FP behavior of varying severity over time. In the present study, 4 days per time period were evaluated to compare the behavior of the LB+ hens with that of the LD hens. In the morning and in the afternoon of each day, the hens were observed for a period of 30 min each (10:00–10:30 h and 15:00–15:30 h). An overview of the behavioral observations performed at different times during the laying period is provided in Table 1. The observed area in each of the four stable compartments measured 1.17 × 1.20 m (length × width) and was located approximately in the middle of the respective compartment. The observed area was regarded representative for the stable compartment, as it included a part of the aviary and of the scratching area, which contained all resources such as litter, feeding through and perches. For an overview of general behaviors, the number of hens performing a certain behavior (ethogram Table 2) was determined using instantaneous scan sampling with a sampling interval of 2 min. Behaviors were interpreted as exclusive events, i.e., each animal was assigned one behavior per scan. When a hen for instance performed comfort behavior, it was not noted whether it occurred in a standing or in sitting position. Location was also not considered separately, i.e., it was not registered whether a hen was standing on a perch, on the tier of the aviary or in the scratching area. Before each scan, the total number of hens present in the observed area was counted. Furthermore, the pecking behavior was recorded in detail by determining the number of pecking bouts for each hen in the observed areas by continuous observations (ethogram Table 3). Repeated pecks at the same conspecific or object were counted as one bout. A bout ended when pecking was stopped for 4 s or when pecking was interrupted by another behavior. All general behavior scans were conducted by one observer; continuous observations of pecking behavior were carried out by two observers. Both observers were trained prior to the evaluation of the videos. Due to phenotypic differences between LB+ (brown feathered) and LD hens (white feathered), the observers were not blinded to hybrid strain.

**Table 1:**
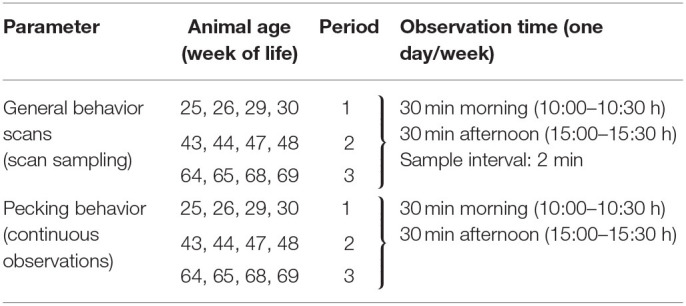
Overview of behavioral observations performed at different times during the laying period.

**Table 2:** Ethogram of general behavior scans [(31) and (35), modified].

**Behavior**	**Description**
Pecking behavior (Peck)	Pecking at conspecifics, ground or objects.
Comfort behavior (Comf)	Includes preening, body shake, wing flap, leg and wing stretch, and tail wag.
Dust bathing (DB)	Manipulation of substrate with the wings, feet, tail, and/or beak while lying in the litter with some or all feathers fluffed.
Scratching (Scra)	Bird standing and scratching repeatedly the litter with one or two feet in a backward movement.
Locomotion (Loc)	Taking at least two consecutive steps.
Stand	Bird is upright and standing on its feet with fully extended legs.
Sit	Bird is upright with its body touching the ground.

**Table 3:** Ethogram of continuous observations of pecking behavior [(31) modified].

**Pecking behavior**	**Description**
Vent Pecking (VP)	Pecks directed to the vent of a conspecific.
Severe Feather Pecking (SFP)	Forceful pecks, sometimes with feathers being pulled out and with the recipient bird moving away.
Gentle Feather Pecking (GFP)	Careful pecks, not resulting in feathers being pulled out and usually without reaction from the recipient bird.
Aggressive Pecking (AP)	Severe and fast, directed mainly at the head and given in a downward direction.
Environmental Pecking (EP)	Pecks directed at any surface, includes ground pecking and object pecking.

### Statistical Analysis

All statistical analyses were performed using the software SPSS Statistics (version 26, IBM, Armonk; NY, USA). To account for the varying number of birds in the observed area at each general behavior scan, data were expressed as proportion of hens performing distinct behaviors. Pecking behaviors in the continuous observations were calculated as bouts per hen and observation interval. The normality of the data was examined using histograms including the Gaussian distribution curve. The Levene procedure was applied to test for homogeneity of variance. To build generalized linear mixed models, data were structured by hybrid × stable compartment (subject) and observation period × week as repeated measures. The models consisted of behaviors of the general behavior scans and continuous observations as target variables, the fixed effects of hybrid, period, the interaction between hybrid and period, and daytime, and the random effect of stable compartment within hybrid. All models were fitted with a normal probability distribution and a log link function, except for environmental pecking, for which an identity link function was applied. For the continuous observations, observer was added as a fixed effect. Fixed effects with *P* > 0.1 (i.e., observer) were excluded in the final models by means of a backward regression procedure. Since vent pecking was not observed at all, it was excluded from statistical analyses. *Post-hoc* pairwise comparisons were adjusted by Bonferroni correction. *P*-values < 0.05 were interpreted to be significant.

## Results

### General Behavior Scans

The proportions of LB+ and LD hens showing the different behaviors, the interaction between hybrid and period, and the effects of hybrid, period and daytime are summarized in Table 4. There was a significant hybrid x period interaction for the proportion of hens pecking [*F*_(2, 89)_ = 6.45, *P* < 0.01], showing comfort behavior [*F*_(2, 89)_ = 6.52, *P* < 0.01] and locomotion [*F*_(2, 89)_ = 9.67, *P* < 0.001], and standing [*F*_(2, 89)_ = 3.92, *P* < 0.05] and sitting [*F*_(2, 89)_ = 10.81, *P* < 0.001]. Pairwise comparison showed that LB+ hens pecked more than LD hens during all periods [*F*_(1, 89)_ = 4.33–19.18, *P* < 0.001– < 0.05]. Within hybrid, a larger proportion of LB+ hens pecked in period 2 and 3 compared to period 1 [*F*_(2, 89)_ = 7.04, *P* < 0.001], whereas there was no difference over time in the LD hens. LD hens showed more comfort behavior than LB+ hens in period 3 [*F*_(1, 89)_ = 7.00, *P* < 0.01]. Within the LB+ strain, comfort behavior occurred to a larger extent in period 1 compared to period 2 and 3 [*F*_(2, 89)_ = 6.05, *P* < 0.01], whereas no such effect was found within the LD strain. A larger proportion of LD hens compared to LB+ hens showed locomotion in period 1 [*F*_(1, 89)_ = 5.84, *P* < 0.05]. Within hybrid, LB+ hens performed more locomotion behavior in period 3 than during the first two observation periods [*F*_(2, 89)_ = 3.61, *P* < 0.05], whereas in the LD hens, locomotion decreased over time [*F*_(2, 89)_ = 11.19, *P* < 0.001]. More LB+ than LD hens were observed standing in period 2 and 3 [*F*_(1, 89)_ = 4.44 and 3.89, *P* < 0.05]. LD hens showed more standing in period 1 than during the remaining observation periods [*F*_(2, 89)_ = 5.11, *P* < 0.05] but no difference was found within the LB+ strain. A larger proportion of LD compared to LB+ hens was sitting in period 2 and 3 [*F*_(1, 89)_ = 76.50 and 179.84, *P* < 0.001]. Within hybrid, LB+ hens showed more sitting behavior in period 1 compared to period 3 [*F*_(2, 89)_ = 3.35, *P* < 0.05], whereas sitting behavior increased throughout the laying period in the LD hens [*F*_(2, 89)_ = 74.38, *P* < 0.001]. Dustbathing and scratching were affected by the main effect of period (i.e., the hens' age) but not by the interaction between hybrid and period. Dustbathing behavior increased over time [*F*_(2, 89)_ = 7.95, *P* < 0.01], whereas scratching decreased [*F*_(2, 89)_ = 65.95, *P* < 0.001] in both hybrids. Daytime affected all observed behaviors, except locomotion. More hens performed comfort behavior, standing and sitting in the morning than in the afternoon [*F*_(1, 89)_ = 8.01–15.60, *P* < 0.001– < 0.01]. In contrast, pecking, dustbathing, and scratching occurred more often in the afternoon [*F*_(1, 89)_ = 17.59–47.04, *P* < 0.001].

**Table 4:** Proportions of conventional layer (LB+) and dual-purpose (LD) hybrids performing distinct behaviors at three times during the laying period (1: 25th−30th, 2: 43rd−48th, 3: 64th−69th week of life) and the day (morning/afternoon).

**Behavior**	**Hybrid**	**Period**			**Daytime**		**P_**hybrid × period**_**	**P_**hybrid**_**	**P_**period**_**	**P_**daytime**_**
		**1**	**2**	**3**	**Morning**	**Afternoon**				
Peck	LB+	38.94	45.55	44.01	33.05	38.27	<0.01	<0.001	ns	<0.001
	LD	30.69	28.74	26.05						
Comf	LB+	16.09	12.29	11.08	15.57	13.13	<0.01	ns	ns	<0.01
	LD	12.60	15.03	19.00						
DB	LB+	0.50	1.17	1.20	0.55	2.95	ns	ns	<0.01	<0.001
	LD	1.02	3.03	3.70						
Scra	LB+	3.38	1.27	0.82	1.44	2.83	ns	ns	<0.001	<0.001
	LD	4.40	1.33	1.61						
Loc	LB+	12.57	12.70	15.27	15.10	14.41	<0.001	ns	<0.01	ns
	LD	20.70	14.43	12.87						
Stand	LB+	24.02	23.83	26.05	24.18	20.45	<0.05	ns	<0.05	<0.001
	LD	23.69	17.83	18.49						
Sit	LB+	4.41	3.11	1.76	10.09	7.87	<0.001	<0.001	<0.05	<0.01
	LD	7.02	19.59	17.98						

### Pecking Behavior

**Figure 1:**
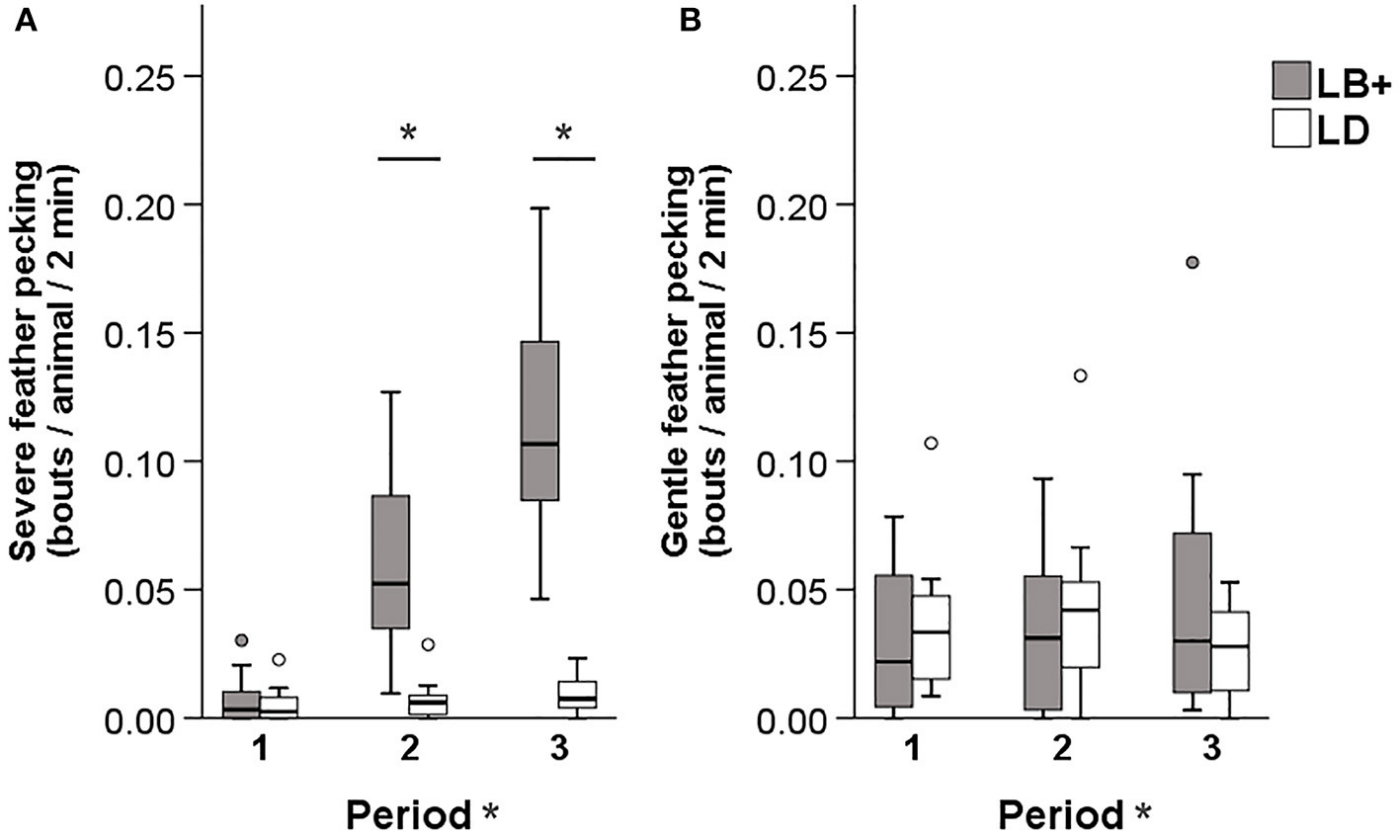
Pecking behavior in conventional layer (LB+) and dual-purpose (LD) hybrids at three times during the laying period (1: 25th−30th, 2: 43rd−48th, 3: 64th−69th week of life). **(A)** Severe feather pecking, **(B)** gentle feather pecking. *Between bars denotes an effect of hybrid (*P* < 0.05). *After “Period” denotes an effect of period (*P* < 0.05). Both for severe feather pecking and gentle feather pecking a hybrid × period interaction was found (*P* < 0.05).

**Figure 2:**
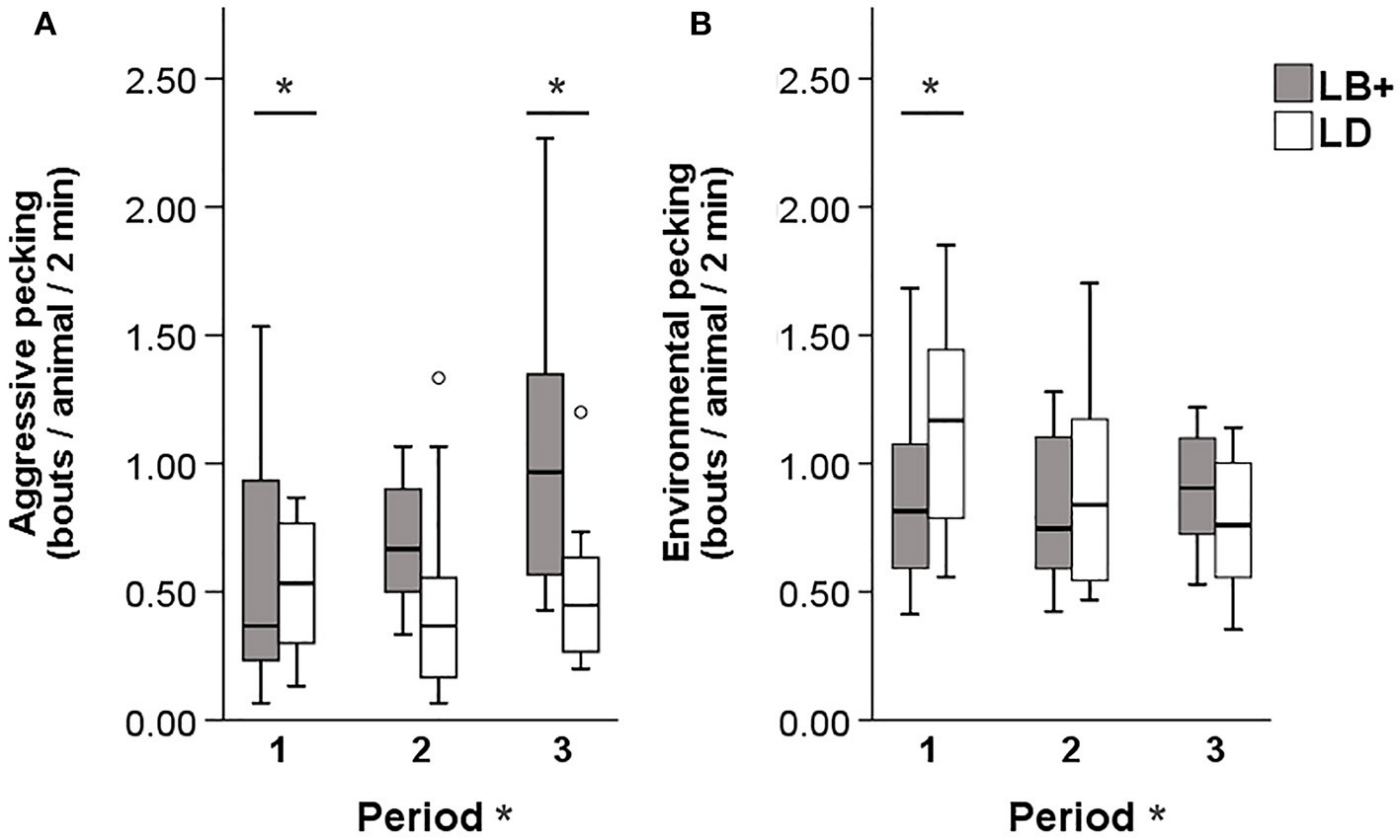
Pecking behavior in conventional layer (LB+) and dual-purpose (LD) hybrids at three times during the laying period (1: 25th−30th, 2: 43rd−48th, 3: 64th−69th week of life). **(A)** Aggressive pecking, **(B)** environmental pecking. *Between bars denotes an effect of hybrid (*P* < 0.05). *After “Period” denotes an effect of period (*P* < 0.05). Both for aggressive pecking and environmental pecking a hybrid × period interaction was found (*P* < 0.05).

A significant hybrid x period interaction was found for the number of severe feather pecking (SFP) [*F*_(2, 89)_ = 6.12, *P* < 0.01] and gentle feather pecking (GFP) events [*F*_(2, 89)_ = 4.55, *P* < 0.05]. Pairwise comparison showed that, while SFP was low in both hybrids during period 1, LB+ hens performed more SFP compared to LD hens in period 2 and 3 [*F*_(1, 89)_ = 29.08 and 21.99, *P* < 0.001]. Within hybrid, an increase of SFP throughout the laying period was observed in the LB+ hens [*F*_(2, 89)_ = 17.82, *P* < 0.001] but not in the LD hens (Figure 1A). There was no difference in GFP between the two hybrids. Within the LB+ strain, more GFP was found in period 3 compared to period 1 and 2 [*F*_(2, 89)_ = 10.78, *P* < 0.001]. In contrast, LD hens showed a peak of GFP in period 2, which was significant compared with period 1 and 3 [*F*_(2, 89)_ = 5.23, *P* < 0.01; Figure 1B]. A significant hybrid x period interaction was also found for the number of aggressive pecking (AP) [*F*_(2, 89)_ = 14.80, *P* < 0.001] and environmental pecking (EP) bouts [*F*_(2, 89)_ = 11.18, *P* < 0.001]. In period 1, LD hens performed more AP compared to LB+ hens [*F*_(1, 89)_ = 20.22, *P* < 0.001], there was no difference between hybrids in period 2, and in period 3, LB+ hens showed more AP than LD hens [*F*_(1, 89)_ = 29.66, *P* < 0.001]. Consequently, AP within the LB+ strain increased throughout the laying period [*F*_(2, 89)_ = 36.81, *P* < 0.001], whereas there was no difference within the LD strain (Figure 2A). Pairwise comparison of EP showed that LD hens were engaged in this behavior to a higher extent than LB+ hens in period 1 [*F*_(1, 89)_ = 4.63, *P* < 0.05] but no difference between hybrids was detected during period 2 and 3. EP in the LD strain decreased from period 1 to period 2 and 3 [*F*_(2, 89)_ = 14.69, *P* < 0.001], whereas there was no difference in EP over time within the LB+ strain (Figure 2B). All pecking behavior, except for gentle feather pecking, was affected by daytime [*F*_(1, 89)_ = 5.23–46.07, *P* < 0.001– < 0.05] with more pecking being observed in the afternoon than in the morning.

## Discussion

The aim of the present investigations was to comparatively assess the behavior with focus on pecking behavior of conventional layer (LB+) and dual-purpose (LD) hybrids during the entire laying period. Therefore, video-based behavioral observations (general behavior scans and continuous observations) were carried out. As expected, most of the observed behaviors, including pecking behavior, were affected by an interaction of hybrid strain and age. In general, the LB+ hens performed more pecking behavior, particularly severe feather pecking (SFP), which increased with age. In contrast, SFP remained exceptionally low in the LD hens throughout the production period. It is important to note that in this study, the hens were observed during the entire laying period (here: 44 weeks) and in a semi-commercial setting. As the social context, the management procedures and the housing system the hens were subjected to can be regarded as representative for many laying farms in Europe, the present results may be applicable to practice directly. On the other hand, however, such a setting only allows for investigating a limited number of groups of animals (here: two stable compartments per hybrid strain). A further limitation of the present study is that the birds were kept in only one pen per hybrid strain during the rearing phase. Environmental factors during rearing, such as the provision or absence of litter, can affect the occurrence of FP behavior during the laying period (36). However, confounding of hybrid strain and rearing environment was kept to a minimum, as both rearing pens were located in the same building, and housing and management conditions, for instance type and quality of litter, were the same for both hybrids.

The general behavior scans of the present study revealed that during all observation periods, the LB+ hens pecked more than the LD hens and within hybrid, pecking behavior of the LB+ hens increased with age. In the LD hens, no difference over time was found. These results are in line with the findings of a previous study by Giersberg et al. (31) on behaviors of LB+ and LD hens in the nest. In this functional area of the barn, more LB+ than LD hens performed total pecking behavior, which was, similar to the present study, defined as the sum of different types of pecking behaviors (SFP, GFP, AP, and EP) (31). Therefore, LD hens seem to be consistently less engaged in pecking activities compared to LB+ hens throughout the laying period. However, due to the different underlying etiology and the resulting consequences of pecking behaviors, it is important to distinguish between different types of pecking behaviors (5), particularly with regard to practical prevention or intervention strategies. Environmental pecking (EP), for instance is characterized by pecks at the ground or objects and is seen as a natural behavior in the context of foraging (37). In contrast, SFP can be regarded as a damaging abnormal behavior, which indicates reduced welfare in both the recipient and the offending bird (5, 9). Therefore, continuous observations were carried out in the present study to determine which type of pecking behavior dominates in the respective hybrid. From the second observation period (43rd−48th week of life) onwards, the LB+ hens performed more SFP compared to the LD hens and within hybrid, an increase of SFP throughout the laying period was observed in the LB+ hens. This confirms the results of Giersberg et al. (6, 34) who found severe feather loss and skin lesions on body regions predisposed to pecking damage in LB+ but not in LD hens. In addition, both the observed increase of SFP within the LB+ strain and the constantly low levels of this behavior within the LD strain reflect the time course of plumage loss assessed by Giersberg et al. (6). The plumage quality of LB+ hens deteriorated with age, whereas this was not the case in LD hens (6). Thus, the present study confirms the assumption that plumage and integument condition are valid indicators for actual SFP behavior, both in conventional layers—as shown previously by Bilčík and Keeling (10)—and in dual-purpose hybrids.

There was no difference between hybrids regarding GFP behavior. Similar results were obtained by van der Eijk et al. (38) when comparing conventional layers with lines that were divergently selected for FP behavior. Hens selected for high FP behavior performed more SFP than hens selected for low FP and unselected control birds, whereas lines did not differ in GFP behavior at adult ages (38). Interpretation of age effects on GFP within hybrid strains remains difficult, as previous studies show ambiguous results. In one study, GFP behavior was inconsistent over time within layer line (38), whereas in another study, GFP decreased with increasing age (39). Thus, a consistent time course of GFP to which the present results could be compared has not been described in the literature. During the first observation period, the LD hens showed more AP compared to the LB+ hens. Within the LD strain, AP behavior remained constant, whereas it increased in the LB+ strain. This lead to a reverse effect of hybrid at the end of the laying period with the LB+ hens showing more AP than the LD hens. The same pattern was found when observing conventional layers and dual-purpose hens in the nest (31). Although AP and SFP result from different underlying motivations (5), it may sometimes be difficult to exactly distinguish between these two behaviors during video observations. In the present study, only pecks given in a downward direction and directed at the head were counted as AP, whereas forceful pecks against the neck and other body parts were recorded as SFP. In video images it may sometimes be difficult to draw a clear line between the different body parts, particularly the head and the neck. This might to some extent explain the increase of SFP and AP with age in the LB+ hens. In addition, the presence of AP behavior in the LD strain further confirms the previous findings regarding plumage condition: feather loss in this strain was only found on the head/neck region (6), which is indicative for AP (10). Similar to AP, the LD hens showed more EP than the LB+ at the beginning of the laying period. Within hybrid strains, EP decreased over time in the LD hens but remained constant in the LB+ hens. As EP is part of the natural foraging and exploration behavior of laying hens (37), these differences are difficult to explain.

However, regarding the other behaviors assessed during the general behavior scans, differences in activity were found, which may also be related to the different types of pecking behavior. For locomotion behavior, a significant hybrid × period interaction was found. Over time, locomotion increased in the LB+ hens, whereas this behavior decreased in the LD hens. In a previous study, a link between activity and FP was identified, with birds performing high levels of FP showing higher levels of locomotion activity compared to birds that perform low levels of FP (16). Furthermore, a larger proportion of LD compared to LB+ hens were observed in a sitting position in period 2 and 3, whilst LB+ showed more sitting in period 1. These findings are inverse to the results on locomotion behavior. The differences in the activity between the LD and LB+ hens over time may be related to their different genetic background. As dual-purpose chickens have a rather compact morphology (33), and they are also breed for meat production, a certain resemblance to broiler chickens can be assumed (40). Behavioral differences between broiler and layer strains may be based in their locomotor ability with layers being more active than broilers (41). Surprisingly, a previous study found that LD hens weighed about 5% less than LB+ hens at 34 weeks of age (33). However, this seemed to be compensated for, as no weight differences were observed between the two hybrids at the end of the laying period (70 weeks of age) (34). In addition, LD hens had about 7% larger body widths and shorter legs compared to LB+ hens (33). This may result in a different relation between body mass and skeletal system, which may lead to an altered locomotor ability in the LD hens. Therefore, the above mentioned decrease of EP behavior in the LD hens may also be due to a general decrease in locomotion and foraging behavior over time.

Concerning comfort behavior, a significant difference between hybrids was found in period 3 with LD hens showing more comfort behavior than LB+ hens. In the LB+ strain, comfort behavior decreased over time. Comfort behavior is a behavioral priority of laying hens with direct effects to animal welfare (18). As feather pecking is a sign of stress, it can be assumed that LB+ hens became agitated with increasing age and hence, comfort behavior decreased. Dustbathing and scratching behavior did not differ between the two hybrid strains. However, it should be noted that these behaviors occurred rarely in both hybrids, which might have overshadowed statistically significant effects.

Daytime effects were found for all observed behaviors, except for locomotion. Comfort behavior, standing and sitting occurred to a larger extent in the morning, while pecking, dust bathing, and scratching were seen more frequently in the afternoon. Laying hens show native circadian patterns of behavior that include egg laying in the morning and dust bathing in the afternoon (42). This is in line with a previous study by Giersberg et al. (31), in which a larger number of LB+ and LD hens stayed in the nests during the first 6 h of the light phase. A diurnal rhythm was also shown for FP behavior, which occurred mainly between 8 and 14 h after lights on (43). Similarly, all pecking behavior observed in the present study, except for GFP, was affected by daytime. In both hybrids, more pecking was observed in the afternoon than in the morning. The present findings reflect the diurnal rhythm of laying hens found previously, with less time spent resting and more time spent performing active behaviors in the afternoon (44).

Housing and management conditions were kept the same for both hybrid strains during the rearing and the laying period. Therefore, behavioral differences, particularly regarding FP, can be likely explained by the genetic differences of the LB+ and LD hens. A genetic background of the development of FP behavior has been suggested in many previous investigations [reviewed by Rodenburg et al. and Channing et al. (5, 44)]. As mentioned earlier, a divergent selection on FP behavior formed the high- and the low FP chicken lines, which are used in fundamental behavioral and physiological research (16, 17, 26, 29, 38). However, it is not clear to which extent those FP traits are present in different commercially available layer hybrids and breeds. In one experiment, conventional layer hybrids (ISA brown) showed more injurious pecking than purebred New Hampshire hens (45). In another experiment, a higher prevalence of FP behavior was found in purebred hens of the Danish landrace compared to two conventional hybrid strains (ISA brown and Lohman selected leghorn) (45). Plumage color seems to be a further genetically determined trait associated with the propensity to develop FP. White hens were less prone to FP than pigmented hens (46, 47). Plumage color might have accounted to a certain extent for the observed hybrid differences in FP in the present study, as the LB+ hens had brown feathers and the LD hens were white. By comparing the LD hens to white feathered conventional layer hybrids in future research, effects of plumage coloration and other strain characteristics could be disentangled.

The present study provides basic information on behavior in general and on different forms of pecking behavior in particular of dual-purpose hens housed in a semi-commercial aviary system. It highlights the behavioral differences between these hens and conventional layer hybrids. As dual-purpose hens show less injurious pecking behavior, they can be kept largely unproblematically with untrimmed beaks and under standard management conditions in loose housing systems. The absence of abnormal behaviors in these hens indicates that they may experience higher levels of welfare than conventional layer hybrids under the given conditions. This major benefit of dual-purpose hybrids should be taken into account when considering alternative approaches to avoid the killing of surplus male animals in laying hen production. Future research should investigate to which extent the present results can be generalized for other commercial farms with slightly different management procedures and housing systems. Further studies are required to investigate whether the observed behavioral benefits are specific for the dual-purpose strain used here or whether they can be reproduced with other dual-purpose hybrids or breeds.

## Data Availability Statement

The raw data supporting the conclusions of this article will be made available by the authors, without undue reservation.

## Ethics Statement

Ethical review and approval was not required for the animal study because the experiments comply with the requirements of the ethical guidelines of the International Society of Applied Ethology (48). All animals were housed according to EU (49) and national law (50, 51). In compliance with European Directive 2010/63/EU Article 1 5.v(f) (52), the present study did not imply any invasive procedure or treatment to the hens.

## Author Contributions

MG, BS, and NK designed the experiments. LR, IZ, and MG performed the data assessment. LR and MG analyzed and interpreted the data, and wrote the paper. BS and NK helped to interpret the results and edited the manuscript. All authors read and approved the final manuscript.

## Conflict of Interest

The authors declare that the research was conducted in the absence of any commercial or financial relationships that could be construed as a potential conflict of interest.
