# Supplementary material for: Pecking Behavior in Conventional Layer Hybrids and Dual-Purpose Hens Throughout the Laying Period
Source: Front Vet Sci. 2021 Apr 23;8:660400. doi: 10.3389/fvets.2021.660400 (PMC8102775; doi:10.3389/fvets.2021.660400)
Supplement: Supplementary file 1 [file Table_1.DOCX]

Supplementary Material

# Environmental conditions for conventional layer (LB+) and dual-purpose (LD) pullets during rearing

The light intensity was measured in six directions at animals’ height in six comparable locations in each pen. The light intensity was measured using a LMT Pocket Lux 2 light meter (LMT Lichtmesstechnik, Berlin, Germany). The mean air flow was measured with a 405-V1 Mini Anemometer (Testo, Lenzkirch, Germany) at animals’ height and in the same locations as the light intensity. CO_2_ and NH_3_ were measured at animals’ height in the middle of each pen using a Dräger accuro pump with indicator tubes (Drägerwerk AG Co. KGaA, Lübeck, Germany). Temperature and relative humidity were measured at animals’ height in the middle of each pen with a PCE-THB 40 data logger (PCE Deutschland GmbH, Meschede, Germany).

**Supplementary Table 1**. Mean light intensity, mean air flow (±SD), CO_2,_ NH_3_, temperature and relative humidity measurements in the rearing pens of conventional layer (LB+) and dual-purpose (LD) pullets.

| Week of age | Pen (hybrid) | Light intensity (LUX) | Air flow (ms/s) | CO_2_ (ppm) | NH_3_ (ppm) | Temperature (`C) | Relative humidity (%) |
| --- | --- | --- | --- | --- | --- | --- | --- |
| 3 | 1 (LB+) | 5.57±0.57 | 0.14±0.12 | 1500 | 5 | 25.9 | 53.4 |
|  | 2 (LD) | 5.43±0.53 | 0.11±0.13 | 1500 | 5 | 25.1 | 55.8 |
| 10 | 1 (LB+) | 5.05±0.69 | 0.09±0.08 | 1500 | 15 | 25.1 | 66.7 |
|  | 2 (LD) | 5.60±1.03 | 0.07±0.06 | 1500 | 15 | 24.6 | 67.0 |
| 17 | 1 (LB+) | 5.34±0.59 | 0.11±0.12 | 1500 | 23 | 20.3 | 69.2 |
|  | 2 (LD) | 5.28±0.96 | 0.08±0.09 | 1500 | 20 | 19.5 | 71.7 |

# Plumage and integument condition of conventional layer (LB+) and dual-purpose (LD) pullets during rearing

Supplementary Table 2. Proportions of conventional layer (LB+) and dual-purpose (LD) pullets with different plumage scores (0 (best) to 4 (worst)) for five body regions obtained by the hands-on scoring method described by Giersberg et al. (2017). N = 50 pullets/hybrid.

| Week of age | Hybrid | Plumage score | Body region | | | | |
| --- | --- | --- | --- | --- | --- | --- | --- |
|  |  |  | Head/neck | Back | Tail | Wing | Breast/belly |
| 3 | LB+ | 0 | 100 | 100 | 100 | 100 | 100 |
|  |  | 1 | 0 | 0 | 0 | 0 | 0 |
|  |  | 2 | 0 | 0 | 0 | 0 | 0 |
|  |  | 3 | 0 | 0 | 0 | 0 | 0 |
|  |  | 4 | 0 | 0 | 0 | 0 | 0 |
|  |  | 0 | 100 | 100 | 100 | 100 | 100 |
|  |  | 1 | 0 | 0 | 0 | 0 | 0 |
|  |  | 2 | 0 | 0 | 0 | 0 | 0 |
|  |  | 3 | 0 | 0 | 0 | 0 | 0 |
|  |  | 4 | 0 | 0 | 0 | 0 | 0 |
| 10 | LB+ | 0 | 100 | 100 | 100 | 100 | 100 |
|  |  | 1 | 0 | 0 | 0 | 0 | 0 |
|  |  | 2 | 0 | 0 | 0 | 0 | 0 |
|  |  | 3 | 0 | 0 | 0 | 0 | 0 |
|  |  | 4 | 0 | 0 | 0 | 0 | 0 |
|  | LD | 0 | 100 | 100 | 100 | 100 | 100 |
|  |  | 1 | 0 | 0 | 0 | 0 | 0 |
|  |  | 2 | 0 | 0 | 0 | 0 | 0 |
|  |  | 3 | 0 | 0 | 0 | 0 | 0 |
|  |  | 4 | 0 | 0 | 0 | 0 | 0 |
| 17 | LB+ | 0 | 100 | 100 | 100 | 100 | 100 |
|  |  | 1 | 0 | 0 | 0 | 0 | 0 |
|  |  | 2 | 0 | 0 | 0 | 0 | 0 |
|  |  | 3 | 0 | 0 | 0 | 0 | 0 |
|  |  | 4 | 0 | 0 | 0 | 0 | 0 |
|  | LD | 0 | 100 | 100 | 100 | 100 | 100 |
|  |  | 1 | 0 | 0 | 0 | 0 | 0 |
|  |  | 2 | 0 | 0 | 0 | 0 | 0 |
|  |  | 3 | 0 | 0 | 0 | 0 | 0 |
|  |  | 4 | 0 | 0 | 0 | 0 | 0 |

Supplementary Table 3. Proportions of conventional layer (LB+) and dual-purpose (LD) pullets with different integument scores (0 (best) to 3 (worst)) for five body regions obtained by the hands-on scoring method described by Giersberg et al. (2017). N = 50 pullets/hybrid.

| Week of age | Hybrid | Plumage score | Body region | | | | |
| --- | --- | --- | --- | --- | --- | --- | --- |
|  |  |  | Head/neck | Back | Tail | Wing | Breast/belly |
| 3 | LB+ | 0 | 100 | 100 | 100 | 100 | 100 |
|  |  | 1 | 0 | 0 | 0 | 0 | 0 |
|  |  | 2 | 0 | 0 | 0 | 0 | 0 |
|  |  | 3 | 0 | 0 | 0 | 0 | 0 |
|  | LD | 0 | 100 | 100 | 100 | 98 | 100 |
|  |  | 1 | 0 | 0 | 0 | 2 | 0 |
|  |  | 2 | 0 | 0 | 0 | 0 | 0 |
|  |  | 3 | 0 | 0 | 0 | 0 | 0 |
| 10 | LB+ | 0 | 100 | 100 | 100 | 100 | 100 |
|  |  | 1 | 0 | 0 | 0 | 0 | 0 |
|  |  | 2 | 0 | 0 | 0 | 0 | 0 |
|  |  | 3 | 0 | 0 | 0 | 0 | 0 |
|  | LD | 0 | 100 | 100 | 98 | 100 | 100 |
|  |  | 1 | 0 | 0 | 2 | 0 | 0 |
|  |  | 2 | 0 | 0 | 0 | 0 | 0 |
|  |  | 3 | 0 | 0 | 0 | 0 | 0 |
| 17 | LB+ | 0 | 100 | 100 | 100 | 100 | 100 |
|  |  | 1 | 0 | 0 | 0 | 0 | 0 |
|  |  | 2 | 0 | 0 | 0 | 0 | 0 |
|  |  | 3 | 0 | 0 | 0 | 0 | 0 |
|  | LD | 0 | 100 | 100 | 98 | 100 | 100 |
|  |  | 1 | 0 | 0 | 2 | 0 | 0 |
|  |  | 2 | 0 | 0 | 0 | 0 | 0 |
|  |  | 3 | 0 | 0 | 0 | 0 | 0 |
